# A Diet Score Assessing Norwegian Adolescents’ Adherence to Dietary Recommendations—Development and Test-Retest Reproducibility of the Score

**DOI:** 10.3390/nu8080467

**Published:** 2016-07-29

**Authors:** Katina Handeland, Marian Kjellevold, Maria Wik Markhus, Ingvild Eide Graff, Livar Frøyland, Øyvind Lie, Siv Skotheim, Kjell Morten Stormark, Lisbeth Dahl, Jannike Øyen

**Affiliations:** 1 National Institute of Nutrition and Seafood Research (NIFES), P.O. Box 2029 Nordnes, 5817 Bergen, Norway; mma@nifes.no (M.K.); mwi@nifes.no (M.W.M.); igr@nifes.no (I.E.G.); lfr@nifes.no (L.F.); oli@nifes.no (Ø.L.); lda@nifes.no (L.D.); joye@nifes.no (J.Ø.); 2 Department of Clinical Medicine, Faculty of Medicine and Dentistry, University of Bergen, P.O. Box 7800, 5020 Bergen, Norway; 3 Regional Centre for Child and Youth Mental Health, Uni Research Health, P.O. Box 7810, 5020 Bergen, Norway; siv.skotheim@uni.no (S.S.); kjell.stormark@uni.no (K.M.S.); 4 Department of Health promotion and development, University of Bergen, P.O. Box 7800, 5020 Bergen, Norway

**Keywords:** FFQ, dietary assessment, adolescents, dietary index, reliability

## Abstract

Assessment of adolescents’ dietary habits is challenging. Reliable instruments to monitor dietary trends are required to promote healthier behaviours in this group. The purpose of this cross-sectional study was to assess adolescents’ adherence to Norwegian dietary recommendations with a diet score and to report results from, and test-retest reliability of, the score. The diet score involved seven food groups and one physical activity indicator, and was applied to answers from a semi-quantitative food frequency questionnaire (FFQ) administered twice. Reproducibility of the score was assessed with Cohen’s Kappa (κ statistics) at an interval of three months. The setting was eight lower-secondary schools in Hordaland County, Norway, and subjects were adolescents (*n* = 472) aged 14–15 years and their caregivers. Results showed that the proportion of adolescents consistently classified by the diet score was 87.6% (κ = 0.465). For food groups, proportions ranged from 74.0% to 91.6% (κ = 0.249 to κ = 0.573). Less than 40% of the participants were found to adhere to recommendations for frequencies of eating fruits, vegetables, added sugar, and fish. Highest compliance to recommendations was seen for choosing water as beverage and limit the intake of red meat. The score was associated with parental socioeconomic status. The diet score was found to be reproducible at an acceptable level. Health promoting work targeting adolescents should emphasize to increase the intake of recommended foods to approach nutritional guidelines.

## 1. Introduction

European adolescents only eat half of the recommended amount of fruits and vegetables and less than two thirds of the recommended amount of milk and milk products. Moreover, they consume more meat and meat products, fats, and sweets than recommended [1]. Despite increased preventive efforts, childhood and adolescent obesity is on the rise in numerous countries, resulting in an increased prevalence of nutrition-related diseases which were previously mainly seen in adults [2]. 

Adolescence is a period that marks the transition from childhood into adulthood, and involves distinct physical, behavioural, and psychological changes [3]. Increases in energy and nutrient requirements, pubertal hormonal changes [4], and increased autonomy [5] are some of the factors that may affect food choices in this group. Dietary and exercise behaviour established in adolescence track into adulthood [6], meaning that unhealthy behaviours could impact adolescents’ present health, as well as their future adult health [7]. The World Health Organization (WHO) recently published a report expressing concern about the negative health trends observed in adolescents, and called for more knowledge about the health behaviour of this group [8]. Accordingly, reliable and user-friendly instruments to monitor dietary trends in adolescents are needed in order to evaluate interventions and to effectively plan and implement preventive and health promoting work. 

All self-reported dietary intake data are prone to errors and assessment of teenagers’ dietary habits can be particularly challenging, as irregular meals, snacking, and meal skipping are common characteristics for this group [9]. Although detailed methods, such as weighed dietary records or recalls are required to assess the intake of energy and single nutrients, a semi-quantitative food frequency questionnaire (FFQ) is a more time and cost-effective way to cover general dietary patterns. A FFQ is, therefore, practical in observational and multidisciplinary study designs, where the number of questions available for each topic may be limited. In addition, answering this type of questionnaire does not require weighing of food or distinct knowledge about brands, which is an advantage when studying adolescents [10,11]. Data from FFQs are useful for calculating scores or indices based on the reported intake of food or food groups. Former studies have used this technique to evaluate adherence to e.g., the Mediterranean diet [12], the Nordic diet [13,14], and a diet to avoid excessive weight gain in pregnancy [15]. Other studies have used the method to account for multiple dietary components and combine them into a single score that measures diet quality. Such scores have been used to measure adherence to dietary guidelines in adults, e.g., in the USA [16], the Netherlands [17], and Australia [18]. In the present study, we developed a diet score based on the Norwegian dietary recommendations, and applied it to a short semi-quantitative FFQ developed for adolescents. The aims were to construct a diet score which might be used to assess adolescents’ adherence to current dietary recommendations, and to report test-retest reliability of the score.

## 2. Materials and Methods 

### 2.1. Study Design and Population

The data in this article are cross-sectional, obtained from a randomized trial (RT) conducted in Bergen, Norway, during spring 2015. Inclusion criteria were girls and boys attending lower-secondary schools (9th grade) with more than two classes at each grade level, who were familiar with the Norwegian language (oral and written). Exclusion criteria were allergy or intolerance to the food or supplements given in the intervention. 

**Figure 1:**
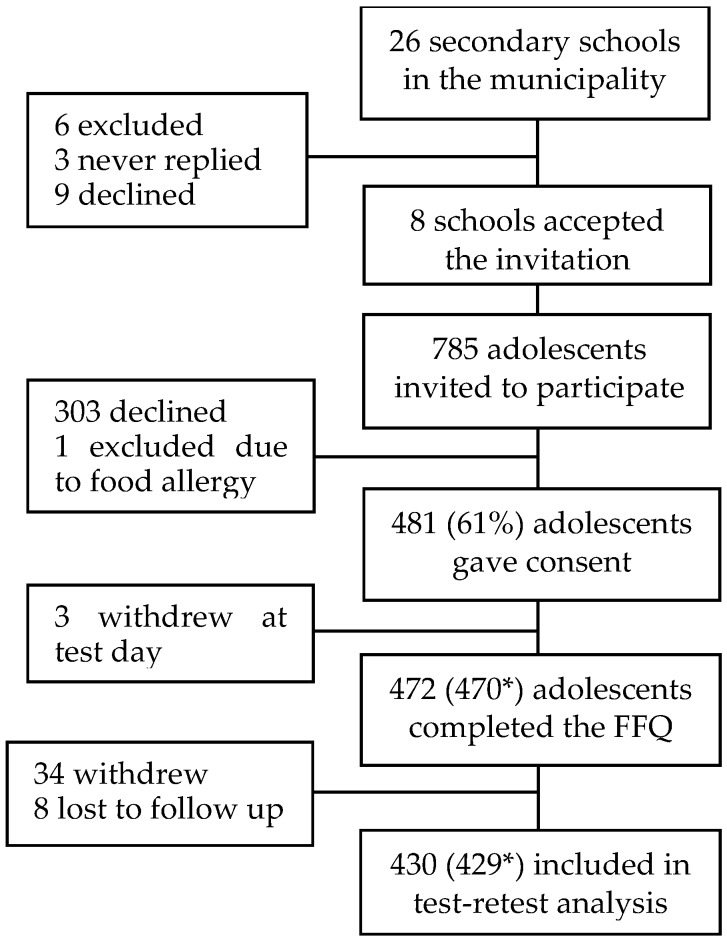
Flowchart showing the study population. * Number of participants with a diet score. FFQ: Food frequency questionnaire.

Initial phone calls were made with twenty-six lower-secondary schools (Figure 1). Three never replied after repeated attempts of contact, six were excluded due to less than three classes at 9th grade, nine schools declined the invitation, and eight schools attended the study. A total of 785 adolescents were attending 9th grade at the time of recruitment and were invited to take part by research staff visiting each classroom giving oral and written information about the study. A total of 481 adolescents (61%) gave their consent to participate, and everyone responding to a FFQ at baseline were included in the analysis (*n* = 472). During the RT, 34 participants withdrew from the trial and eight were lost to follow up, resulting in test-retest data from 430 participants. Participants with missing data in one or more of the questions in the diet score were excluded from the analyses where the score was used (*n* = 2 and *n =* 1 participants in first and second administration of the FFQ, respectively). A web-based questionnaire was also sent to the parents/caregivers by e-mail, and 370 questionnaires (78%) were completed (data not shown in Figure 1). This study was conducted according to the guidelines laid down in the Declaration of Helsinki and all procedures involving human subjects were approved by the Norwegian Data Protection Official for Research (project number: 41030) and registered in ClinicalTrials.gov (NCT02350322). Written informed consent was obtained from all subjects and their caregivers, whom could withdraw from the study at any time without reason. 

### 2.2. Online FFQ

Participants completed a short semi-quantitative retrospective FFQ at school, three months apart. The online FFQ was created using Qualtrics.com^®^ (Provo, UT, USA). Research staff were present to answer questions, and provided participants with their personal username and password to login to the questionnaire. Participants were instructed to give an average of their intake the past three months, and the questionnaire was completed within 15 min. The FFQ should cover two aspects: (1) the participants’ habitual diet besides the intervention; and (2) the adherence to the dietary recommendations that are measurable in a semi-quantitative FFQ. Thus, the FFQ assessed the frequency of eating seafood and red meat for dinner, fruits, vegetables, juice and smoothies, sweets and sugary soda, whole grain content in bread/cereals/crispbreads, and drinking water, in addition to physical activity. The participants’ age, weight, height, and gender were self-reported in the FFQ. Body mass index (BMI) was calculated as weight in kilograms divided by the square of the height in meters (kg/m^2^). To classify weight status, Cole’s age and sex-specific BMI cut off points for underweight [19], and overweight and obesity [20] for adolescents (14.5 years) were used. The cut offs for thinness are 16.7 for boys and 17.2 for girls. For overweight the cut offs are 23.0 and 23.7 for boys and girls respectively, and for obesity they are 28.0 and 28.9 for boys and girls, respectively. A detailed overview of the questions and response categories in the FFQ is shown in Table S1.

### 2.3. Development of the Diet Score

The diet score is created to measure the participants’ adherence to eight out of 13 specific nutritional recommendations given by the Norwegian Directorate of Health [21]. Five recommendations were excluded from the score because they were not readily measureable in a semi-quantitative FFQ. One was a summary of all the recommendations, two were too general: ‘Obtain balance between energy intake and expenditure’ and ‘Supplements may be necessary for some groups’ and two could not be accurately measured in a short FFQ: ‘Select edible oils and soft margarines’ and ‘Limit the intake of salt’.

Eight indicators corresponding to the eight included recommendations were made, of which seven covered food groups and one covered physical activity (Table 1). For five of the indicators, cut-offs scored the answers from the FFQ directly with: 0 points = not adhering, or 1 point = adhering to recommendations. Since the indicators for fruits and vegetables and added sugar involved more than one question in the FFQ, data from these questions were given indices (Table 2 and Table 3). The indices were summarized into a total index for each indicator before they were scored with 0 or 1 point. The total diet score for a participant was calculated by summarizing the points from the indicators. Hence, a participants’ diet score sum reflected the number of recommendations he or she was found to comply. No further credit was given, nor were any points deducted if the number of servings exceeded the cut-offs for any of the indicators. For some analyses, the diet score was trichotomized in order to make categories equivalent to a Low (0–3 points), Moderate (4–5 points), or High (6–8 points) adherence to the dietary recommendations.

### 2.4. Caregivers’ Questionnaire

A web-based questionnaire sent to the parents/caregivers assessed demographic and socioeconomic information, such as parental educational level and total household income. The response categories for educational level (separate for mother and father) were ‘elementary/lower secondary school (10 years or less)’, ‘vocational/upper secondary school (13 years or less)’, ‘high school/matriculation (13 years or less)’, ‘college/university (three years or less)’, or ‘college/university (four years or more)’. In the analysis, educational levels were dichotomized into ‘lower (13 years or less)’ or ‘higher (College/university)’. Household income was given the response categories ‘<200,000 NOK’, ‘201,000–349,999 NOK’, ‘350,000–549,999 NOK’, ‘550,000–749,999 NOK’, ‘750,000–999,999 NOK’, ‘1,000,000–1,249,999 NOK’, ‘1,250,000–2,000,000 NOK’ and ‘>2,000,000 NOK’ (100 NOK = approximately 10€/11$). In the analysis, the categories were combined into ‘<200,000–749,999’ NOK, ‘750,000–1,249,999 NOK’, and ‘1,250,000–>2,000,000 NOK’. 

### 2.5. Test-Retest 

Of the 472 participants who answered the FFQ at baseline, 430 (91%) also completed the FFQ three months later, and these were included in the test-retest analysis (Figure 1). The same research staff administered the login and were present to answer questions on both occasions. Since the purpose of the FFQ was to measure the participants’ background diet (besides the intervention), respondents were instructed not to include the experimental food or supplements when answering the second time. 

### 2.6. Statistical Analyses 

Statistical analyses were performed using the Statistical Package for the Social Sciences (IBM^®^ SPSS^®^ Statistics version 22, IBM Corporation, Kolbotn, Norway) and STATA^®^ version 14.0 for Windows (StataCorp LP, Metrika Consulting AB, Västervik, Sweden). Differences in participant characteristics across the trichotomized diet score were analysed with Pearson’s chi-square (*X*^2^) test for categorical variables, and with one-way ANOVA for ≥1 continuous variables. Cohen’s kappa measure of agreement (κ) was used to assess how consistently the dichotomized indicators categorized subjects, and Cohen’s weighed κ assessed the consistency of the diet score in order to distinguish between different degrees of disagreement [22]. A κ > 0.04 was interpreted as acceptable agreement [23]. A *p*-value of < 0.05 was considered statistically significant.

## 3. Results

### 3.1. Participant Characteristics

Table 4 gives a summary of the overall participant characteristics and according to their trichotomized diet score. The participants mean age was 14.5 years (SD 0.34) and the male/female ratio was 224/248. Almost 80% of the participants had a BMI within the normal range. The participants were categorized according to the diet score into Low (*n* = 127), Moderate (*n* = 226), or High (*n* = 117) adherence to the dietary recommendations, which represented 27%, 48%, and 25% of the sample, respectively. There were significant differences between the groups in the mothers’ (*p* = 0.001) and the fathers’ (*p* = 0.018) education levels and family income levels (*p* = 0.002). Higher parental income/education level was associated with higher diet scores in the participants. 

### 3.2. Adherence to Dietary Recommendations

**Figure 2:**
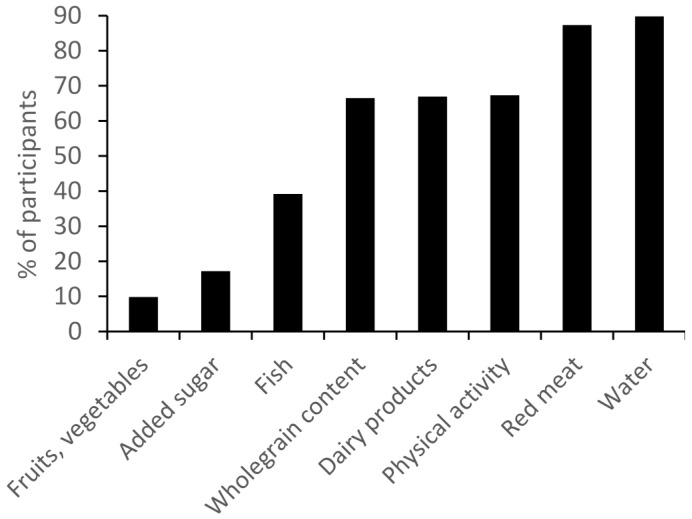
Percentages of participants *(n* = 470) who were found to comply with the different recommendations in the diet score.

**Figure 3:**
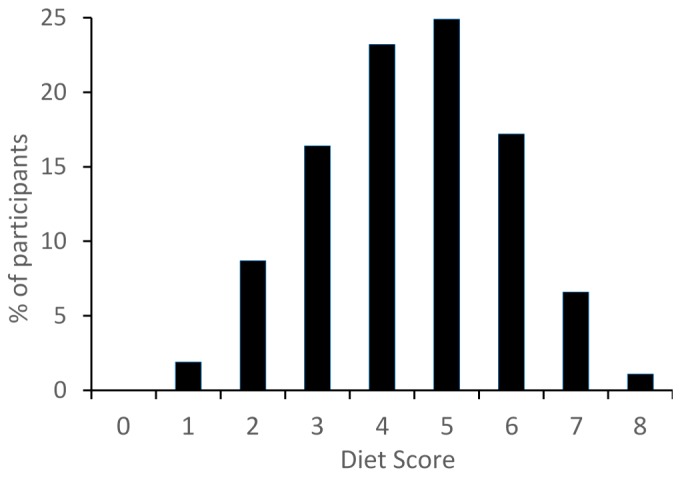
Overview of the percentage of participants (*n* = 470) with diet score sums from zero to eight points.

Figure 2 shows the percentages of participants who were found to comply with the different recommendations in the diet score. Fruits and vegetables, added sugar, and fish were recommendations which most participants failed to comply (10%, 17%, and 39% of participants complied, respectively), whereas recommendations concerning red meat and water had the highest compliance among participants (87% and 90% complied, respectively). Figure 3 shows the percentage of participants achieving a diet score sum between zero and eight points. The majority of participants had moderate diet scores of four or five points (23% and 25% of the sample, respectively) and none of the participants obtained zero points, indicating that everybody adhered to at least one recommendation.

Agreement of the diet score and the dichotomized indicators between the two FFQs is shown in Table 5. The real percentage agreement for the Diet Score (87.6%) and the indicators (74.0%–91.6%) exceeded expected agreement for all parameters, and Cohen’s κ was > 0.4 for all parameters, except red meat (κ = 0.249). 

## 4. Discussion

In the present study, we found that less than 40% of the participants had a habitual diet according to the recommendations for fruits, vegetables, fish, and added sugar. Additionally, we found that the diet score categorized participants consistently according to the number and types of recommendations they adhered to (κ > 0.4), indicating the method to be reproducible at an acceptable level [23]. 

The present study has a number of strengths and limitations. The strengths were the large study population, and the relatively large number of participants who completed the FFQ twice. In addition, the diet score was constructed from already-defined recommendations. This limits the risk of subjectivity in which food components to include and where to set the cut-offs [24]. The three-month interval is a strength, because shorter intervals increase the risk of respondents recalling their previous answers in the test-retest [25], still, it is too short for any real changes in diet to occur. The score is, to our knowledge, less comprehensive than other scores and indices established to date, which makes it easy to include in questionnaires and statistics. 

The main limitation of this study is that the school lunch intervention between the FFQs could have biased reliability. One cannot exclude that the meals given in the study could have substituted for other food items and, thereby, changed the diet. Therefore, the reliability of the score should be interpreted with caution. However, logically the intervention could only have affected reliability negatively (type 2 error). Since we found comparable agreement of the questions in the FFQ (Table S2) and the diet score as to previous studies, this might indicate a robustness of the score. Moreover, the intervention was not very comprehensive. The experimental groups involved giving participants omega-3 supplements or school meals, three times a week during their lunch break. In addition, the FFQ was constructed to assess the participants’ background diet, not their adherence to the intervention. Other limitations in this study were that although the recommendations advise an intake of low-fat dairy products and four portions of wholegrain products per day, the score assessed dairy and whole grain products in general to avoid questions about brands and quantities. Moreover, the Norwegian population is recommended to limit the intake of red meat and to consume primarily lean meat, whereas the diet score only considers the frequency of intake and not the content of fat. Another limitation is that assessment of physical activity was covered with one question only, and a ceiling effect was apparent, indicating that the maximum of four hours per week was insufficient. Other lifestyle aspects, such as sleep duration and sleep quality, were not included in the score because the score included factors communicated in the dietary recommendations exclusively. Generalizability can be questioned as roughly 1/3 of the invited schools, and 61% of the adolescents attending 9th grade at these schools participated. Thus, there is a risk that this sample had healthier habits compared to the representative adolescent population in Bergen [26]. Finally, a key issue of this study is that although reliability of the questionnaire (Table S2) and the diet score was considered acceptable, the validity still remains unexplored. Dietary self-report data always bears the risk of over- or under-reporting [27,28]. For instance, pooled results from five studies in adults from the US using FFQ validated against recovery biomarkers (doubly-labelled water) showed that the average rate of under-reporting for energy intake with FFQ was 30% (range, 24%–32%) [29]. 

To our knowledge, describing dietary intake by assessing adherence to a set of dietary guidelines has previously been done twice in adolescents [30,31]. Golley et al. [30] established the Dietary Guideline Index for Children and Adolescents (DGI-CA) using the 2003 Australian dietary guidelines for children and adolescents. The DGI-CA comprised 11 indicators, one reflecting dietary variety, nine reflecting dietary adequacy and quality, and one reflecting dietary moderation. Similar to our study, the total DGI-CA score was the sum of the 11 indicators where a higher score reflected greater adherence to the guidelines. Comparable to our results, this study also found low intakes of fruits and, particularly, vegetables in their sample of 12–16 year olds. In the Healthy Lifestyle in Europe by Nutrition in Adolescence study [31], 1593 adolescents (12.5–17.5 year olds) completed two 24 h recalls using the HELENA-DIAT software. Their intakes were compared with the Optimal Mixed Diet and the Food Guide Pyramid. They found that about 40% of the adolescents met the recommendations for fruits and vegetables, which is higher than the results from our study. They also found that recommendations for meat and sugar were greatly exceeded, which is in line with our results. 

In the present study, as many as 77% and 92% of the adolescents failed to meet the recommend two and three portions of fruits and vegetables per day, respectively, and 59% and 50% ate even less than one portion of fruits and vegetables per day (Table S1). This is of concern as it is established that fruits and vegetables are great sources of vitamins, minerals, antioxidants, and fibre, in addition to being low in energy and protective against disease and mortality [32]. Low intakes of fruits and vegetables among adolescents have been reported previously, for instance in the WHO-initiated ‘Global school-based student health survey’ (GSHS) [33]. This survey found that 69% of European 15 year-olds reported to not eat fruit daily. Hence, this seems to be a general challenge among European adolescents. Among the participants in the present study, only 17% were found to adhere to the recommendation to limit the intake of added sugar. Too high intakes of sugar-sweetened beverages has been put forward as a problem among the adolescent population worldwide [34], and is associated with increased energy intake, weight gain, and lower intakes of milk, calcium, and other nutrients [35]. Results from the GSHS survey suggests that 25% of European adolescents (15 year olds) reported to drink soft drinks at least on a daily basis [33]. The explanation for the high proportion of participants (90%) adhering to the recommendation regarding water drinking, could be that the cut off for adhering was ‘once a day’ making it rather undemanding to comply with this recommendation. Still, it is noteworthy that as many as 10% was found to not adhere to this recommendation, which might imply consumption of other beverages at the expense of water on a daily basis. It is also noteworthy that as many as 26% of the adolescents reported to eat fish for dinner less than once a month (Table S1), which is below half of the recommended amount. We found that the adolescents’ diet score was associated with their families’ socioeconomic position. This is in accordance with existing literature [30,36], thus, further confirming the diet score’s ability to capture diet quality. Evidence from a previous Norwegian study indicates that parents from lower social classes are less knowledgable about dietary guidelines compared to parents from higher classes [37]. It may, therefore, be wise to tailor nutritional interventions based on socioeconomic factors when targeting children and adolescents in the future.

In the present study, the test-retest agreement ranged from 74% to 92% for the diet score and its indicators, and agreement was also high for the specific questions in the FFQ (67%–98%, Table S2). Previous studies have generally reported lower percentage agreements in adolescents [38,39,40]. Øverby et al. [38] included 58 Norwegian adolescents (14–15 years) who completed a 131-item FFQ four weeks apart. They reported lower percentages for correct classifications into quartiles for food groups (36%–55%) compared to our study. In another study of 48 Danish adolescents aged 13–15 years, who completed a 145-item FFQ four weeks apart, Bjerregaard et al. [39] also found lower agreement compared to our study in questions regarding frequently consumed food items (34%–53%), rarely consumed food items (55%–70%) and less healthy food items (32%–58%). However, these studies used smaller sample sizes and longer questionnaires compared to the present study, which makes comparisons difficult. Still, Xia and colleagues [40] used a slightly shorter FFQ (81-items) and a larger sample size (168 girls, 12–18 years) than these studies and, yet, they also reported slightly lower percentage agreement when categorizing food groups into quartiles (65%–84%) compared to our study. In the present study, Cohen’s κ values were above 0.4 (except for one diet score indicator and four FFQ questions (Table S2)) which has been suggested to be adequate for reproducibility studies in adults [23]. Previous studies using correlation coefficients have reported slightly higher agreement, although direct comparison with Cohen’s κ is difficult. Øverby et al. [38], reported median Spearman coefficients for food groups between 0.52 and 0.67, and Xia et al. [40] reported intra-class correlations between 0.58 and 0.73 for food groups. However, Bjerregaard et al. [39] found that Cohen’s weighed κ ranged from 0.23 to 0.71 for food groups, which is equivalent to our study findings. Importantly, factors, such as the number of food items and categories in the FFQ, sample size, and time period between FFQ administrations could affect the level of agreement. Cohen’s κ was used to assess reliability in the present study because it takes agreement occurring by chance into account. Furthermore, it has been argued that it is superior to correlation coefficients in its ability to detect agreement between two (possibly different) measures, and not merely the degree of linear associations [41]. Overall, the reproducibility found in the present study was acceptable and comparable to former studies despite some differences in sample size, methodology, and design of the FFQs. 

As commented in the review by Waijers et al. [42], there are some key issues in the construction and composition of dietary scores which could influence their usefulness and validity as measures of dietary patterns. For instance, in the present study, each of the eight diet score indicators reflected one recommendation and had no ranking of importance. This is consistent with how the recommendations are communicated by the authorities and with the methodology used in previous studies using scores and indices. Still, whether this method is ideal remains uncertain. Nevertheless, the strengths with dietary scores and indices are their ability to account for the complexity of food patterns, and examine them prospectively in relation to various outcomes, such as type 2 diabetes [43], mortality [44], or mental health [45]. Thus, scores and indices can provide a broader understanding of nutritional causes for disease than any individual nutrient or food item [46]. Importantly, the diet score was not constructed to differentiate between individuals in a clinical trial, but should be used to classify group differences with respect to dietary patterns in epidemiological research. The mentioned traits with the score, such as online administration and that the eight questions comprising the score are easily included in almost any kind of questionnaire, makes it user friendly. The majority of European countries have some form of dietary recommendations, and they usually include advice about intakes of red meat, sugar, fish, whole grains, fruits, and vegetables. Hence, it is the authors’ belief that the diet score, or at least its principle, could be a useful tool in dietary research in the future. 

## 5. Conclusions

In conclusion, the diet score was found to be reproducible at an acceptable level, and in agreement with previous studies in adolescents. The results suggest that most adolescents in our sample are not in compliance with the recommendations for fruits, vegetables, fish, and added sugar. Future health-promoting work targeting this age group should focus on these foods so that they approach recommended levels.

## Figures and Tables

**Table 1:** The dietary recommendations, corresponding indicators, and cut offs for development of the diet score.

	Dietary Recommendation	Indicator	Cut-Off	Points
1	Eat at least five portions of fruits and vegetables every day (maximum one glass of juice can be included as one portion)	Fruits, vegetables, juice and smoothies (total index) ^a^	<5.0	0
			≥5.0	1
2	Eat at least four whole grain products every day	Wholegrain content in bread, cereals, crisp breads	<50%	0
			≥50%	1
3	Eat fish corresponding to 2–3 dinner servings a week	Fish (dinner servings)	≤1/week	0
			≥2/week	1
4	Choose lean meat and meat products and limit your intake of red- and processed meat	Red meat (dinner servings)	≤3/week	1
			≥4/week	0
5	Low-fat dairy products should be a part of the daily diet	Dairy products (portions)	≤6/week	0
			≥1/day	1
6	Limit your intake of added sugars	Sweets and sugary soda (total index) ^b^	>0.2	0
			≤0.2	1
7	Water is the recommended beverage	Water (frequency)	≥1/day	1
			<1/day	0
8	Do some form of physical activity for at least 30 min a day	Physical activity (hours)	≥4/week	1
			<4/week	0
		Maximum reachable score:		8

^a^ Points calculated from indices (Table 2); ^b^ Points calculated from indices (Table 3).

**Table 2:** Indices given to ordinal data from the FFQ to score fruit, vegetable, juice, and smoothie intake.

Indices for Fruit and Vegetable Intake							
	Never/Seldom	1–3 Portions/Week	4–6 Portions/Week	1 Portion/Day	2 Portions/Day	3 Portions/Day	≥4 Portions/Day
Fruits	0	0.3	0.7	1	2	3	4
Vegetables	0	0.3	0.7	1	2	3	4
Juice ^a^	0	0.1	0.3	0.5	0.5	0.5	0.5
Smoothies ^a^	0	0.1	0.3	0.5	0.5	0.5	0.5

^a^ The indices given to response categories for juice and smoothies stopped at 0.50 to take into account that maximum one glass of juice/smoothies could be included as one portion per day.

**Table 3:** Indices given to ordinal FFQ data to score sugar intake.

	Index
Sweets at school	
Never/seldom	0
1–2 times/week	0.2
3–4 times/week	0.5
Every day	1
Sweets at home	
Never/seldom	0
1–2 times/week	0.2
3–4 times/week	0.5
5–6 times/week	0.8
Every day	1
Sugary soft drinks	
Never/seldom	0
1–3 times/week	0.3
4–6 times/week	0.7
Every day	1
2 times/day	2
3–4 times/day	3.5
>5 times/day	5

**Table 4:** Characteristics of the participants and their parents shown for all participants, and according to the participants’ trichotomized diet scores. *p*-Value indicates differences across diet score categories. Data given as *n* (%) if not otherwise indicated.

	*n*	All (*n* = 472)	Diet Score			*p*-Value ^a^
			Low (*n* = 127)	Moderate (*n* = 226)	High (*n* = 117)	
Age, years (Mean SD)	472	14.6 (0.3)	14.5 (0.4)	14.6 (0.3)	14.6 (0.3)	0.143
Height, cm (Mean SD)	447	169 (0.09)	168 (0.1)	170 (0.1)	169 (0.1)	0.239
Weight, kg (Mean SD)	459	57 (10.6)	56 (10.8)	57 (11.0)	58 (10.1)	0.620
Gender	472					0.053
Girls		248 (52.5)	77 (60.6)	107 (47.3)	63 (53.8)	
Boys		224 (47.5)	50 (39.4)	119 (52.7)	54 (46.2)	
**BMI category ^b^**	435					0.835
Underweight		56 (12.9)	17 (14.4)	28 (13.7)	11 (9.9)	
Normal weight		345 (79.3)	91 (77.1)	161 (78.5)	92 (82.9)	
Overweightor obese		34 (7.8)	10 (8.5)	16 (7.8)	8 (7.2)	
**Parental education ^c^**						
Mother:	369					0.001
Lower		107 (28.9)	39 (42.9)	51 (27.3)	17 (18.7)	
Higher		263 (71.1)	52 (57.1)	136 (72.7)	74 (81.3)	
Father:	370					0.018
Lower		151 (40.8)	49 (53.3)	71 (38.0)	31 (34.4)	
Higher		219 (59.2)	43 (46.7)	116 (62.0)	59 (65.6)	
**Family income in NOK ^d^**	368					0.002
<200,000–749,999		76 (20.7)	25 (27.5)	39 (21.0)	12 (13.3)	
750,000–1,249,999		190 (51.6)	55 (60.4)	88 (47.3)	46 (51.1)	
1,250,000–>2,000,000		102 (27.7)	11 (12.1)	59 (31.7)	32 (35.6)	

**^a^** One-way ANOVA test (continuous variables) and Pearson’s chi-square test (*X*^2^) (categorical variables); **^b^** BMI, Body mass index. Cole’s criteria for underweight, overweight and obesity according to gender; **^c^** Lower education ≤ education at the elementary, high school or vocational school. Higher education: Having attended college or university education; **^d^** NOK, Norwegian kroner (100 NOK = approximately 10€/11$).

**Table 5:** Test-retest agreement of the diet score and the dichotomized scores of the indicators.

Diet Score and Indicators	*n*	κ Measure of Agreement	*p*-Value	Expected Agreement (%)	Agreement (%)
Diet Score	429	0.465 *	<0.001	76.8	87.6
Fruits, vegetables	429	0.493 *	<0.001	83.4	91.6
Wholegrain	430	0.532 *	<0.001	55.3	79.1
Seafood dinner	430	0.454 *	<0.001	52.3	74.0
Red meat	430	0.249	<0.001	77.7	83.3
Dairy products	430	0.469 *	<0.001	57.0	77.2
Added sugar	430	0.573 *	<0.001	70.6	87.4
Water	430	0.443 *	<0.001	81.6	89.8
Physical activity	430	0.552 *	<0.001	57.0	80.7

* = Acceptable agreement (κ > 0.4) [23].

## Supplementary Materials

The following are available online at http://www.mdpi.com/2072-6643/8/8/467/s1, Table S1: Frequency of eating various food shown for all participants and according to the participants Diet Score. *p*-Value indicates differences across Diet Score categories. Table S2: Test-retest agreement of questions in the FFQ.
