# Supplementary material for: A Diet Score Assessing Norwegian Adolescents’ Adherence to Dietary Recommendations—Development and Test-Retest Reproducibility of the Score
Source: Nutrients. 2016 Jul 29;8(8):467. doi: 10.3390/nu8080467 (PMC4997380; doi:10.3390/nu8080467)
Supplement: Supplementary file 1 [file nutrients-08-00467-s001.docx]

Supplementary Materials: A Diet Score Assessing Norwegian Adolescents’ Adherence to Dietary Recommendations—Development and Test-Retest Reproducibility of the Score

Katina Handeland, Marian Kjellevold, Maria Wik Markhus, Ingvild Eide Graff, Livar Frøyland, Øyvind Lie, Siv Skotheim, Kjell Morten Stormark, Lisbeth Dahl and Jannike Øyen

**Table S1.** Frequency of eating various food shown for all participants and according to the participants Diet Score. *p*-Value indicates differences across Diet Score categories.

| **Food Items** | ***n*** | **Frequency of Consumption** | **All  (*n* =472)** | **Diet Score** | | | ***p*-Value ^c^** |
| --- | --- | --- | --- | --- | --- | --- | --- |
|  |  |  |  | **Low  (*n* = 127)** | **Moderate  (*n* = 226)** | **High  (*n* = 117)** |  |
|  |  |  | ***n* (%)** | ***n* (%)** | ***n* (%)** | ***n* (%)** |  |
| Seafood (dinner) ^a^ | 472 |  |  |  |  |  | <0.001 |
|  |  | “Never” | 4 (0.8) | 4 (3.1) | 0 (0) | 0 (0) |  |
|  |  | <1 time/month | 23 (4.9) | 14 (11.0) | 8 (3.5) | 1 (0.9) |  |
|  |  | 1–3 times/month | 97 (20.6) | 50 (39.4) | 43 (19.0) | 4 (3.4) |  |
|  |  | 1 time/week | 163 (34.5) | 55 (43.3) | 98 (43.4) | 10 (8.5) |  |
|  |  | 2–3 times/week | 181 (38.3) | 4 (3.1) | 77 (34.1) | 98 (83.8) |  |
|  |  | ≥4 times/week | 4 (0.8) | 0 (0) | 0 (0) | 4 (3.4) |  |
| Seafood (spread) | 472 |  |  |  |  |  | <0.001 |
|  |  | “Never” | 115 (24.4) | 45 (35.4) | 51 (22.6) | 18 (15.4) |  |
|  |  | “Seldom” | 151 (32.0) | 46 (36.2) | 71 (31.4) | 34 (29.1) |  |
|  |  | 1–3 times/month | 102 (21.6) | 21 (16.5) | 61 (27.0) | 19 (16.2) |  |
|  |  | 1 time/week | 51 (10.8) | 6 (4.7) | 26 (11.5) | 19 (16.2) |  |
|  |  | 2 times/week | 28 (5.9) | 9 (7.1) | 8 (3.5) | 11 (9.4) |  |
|  |  | 3–5 times/week | 21 (4.4) | 0 (0) | 8 (3.5) | 13 (11.1) |  |
|  |  | ≥6 times a week | 4 (0.8) | 0 (0.0) | 1 (0.4) | 3 (2.6) |  |
| Dairy products ^a^ | 472 |  |  |  |  |  | <0.001 |
|  |  | “Never/seldom” | 7 (1.5) | 4 (3.1) | 2 (0.9) | 1 (0.9) |  |
|  |  | 1–3 times/week | 45 (9.5) | 28 (22.0) | 13 (5.8) | 3 (2.6) |  |
|  |  | 4–6 times/week | 104 (22.0) | 52 (40.9) | 43 (19.0) | 9 (7.7) |  |
|  |  | Every day | 152 (32.2) | 25 (19.7) | 82 (36.3) | 45 (38.5) |  |
|  |  | 2 times/day | 75 /15.9) | 6 (4.7) | 40 (17.7) | 28 (23.9) |  |
|  |  | 3–4 times/day | 89 (18.9) | 12 (9.4) | 46 (20.4) | 31 (26.5) |  |
| Wholegrain content in bread ^a^ | 472 |  |  |  |  |  | <0.001 |
|  |  | “I don’t eat bread/crispbread” | 7 (1.5) | 6 (4.7) | 1 (0.4) | 0 (0) |  |
|  |  | Finely ground (0%–25% wholegrain) | 19 (4) | 11 (8.7) | 8 (3.5) | 0 (0) |  |
|  |  | Medium dark (25%–50% wholegrain) | 132 (28) | 73 (57.5) | 56 (24.8) | 2 (1.7) |  |
|  |  | Dark (50%–75% WG) | 252 (53.4) | 33 (26.0) | 134 (59.3) | 84 (71.8) |  |
|  |  | Extra dark (75%–100% wholegrain) | 62 (13.1) | 4 (3.1) | 27 (11.9) | 31 (26.5) |  |
| Red meat ^a^ | 472 |  |  |  |  |  | <0.001 |
|  |  | “Never” | 6 (1.3) | 3 (2.4) | 2 (0.9) | 1 (0.9) |  |
|  |  | <1 time/month | 11 (2.3) | 4 (3.1) | 7 (3.1) | 0 (0) |  |
|  |  | 1–3 times/month | 35 (7.4) | 15 (11.8) | 13 (5.8) | 7 (6.0) |  |
|  |  | 1 time/week | 110 (23.3) | 23 (18.1) | 52 (23.0) | 35 (29.9) |  |
|  |  | 2–3 times/week | 250 (53.0) | 55 (43.3) | 119 (52.7) | 74 (63.2) |  |
|  |  | ≥4 times/week | 60 (12.7) | 27 (21.3) | 33 (14.6) | 0 (0) |  |
| White meat | 472 |  |  |  |  |  | 0.292 |
|  |  | “Never” | 5 (1.1) | 3 (2.4) | 1 (0.4) | 1 (0.9) |  |
|  |  | <1 time/month | 28 (5.9) | 11 (8.7) | 10 (4.4) | 7 (6.0) |  |
|  |  | 1–3 times/month | 108 (22.9) | 35 (27.6) | 51 (22.6) | 22 (18.8) |  |
|  |  | 1 time/week | 174 (36.9) | 42 (33.1) | 86 (38.1) | 44 (37.6) |  |
|  |  | 2–3 times/week | 145 (30.7) | 31 (24.4) | 73 (32.3) | 41 (35.0) |  |
|  |  | ≥4 times/week | 12 (2.5) | 5 (3.9) | 5 (2.2) | 2 (1.7) |  |
| Fruits ^a,b^ | 471 |  |  |  |  |  | <0.001 |
|  |  | “Never/seldom” | 37 (7.9) | 22 (17.3) | 15 (6.6) | 0 (0) |  |
|  |  | 1–3 portions/week | 153 (32.5) | 61 (48.0) | 67 (29.6) | 25 (21.4) |  |
|  |  | 4–6 portions/week | 89 (18.9) | 27 (21.3) | 47 (20.8) | 14 (12.0) |  |
|  |  | 1 portion/day | 84 (17.8) | 12 (9.4) | 48 (21.2) | 24 (20.5) |  |
|  |  | 2 portions/day | 66 (14) | 4 (3.1) | 38 (16.8) | 24 (20.5) |  |
|  |  | 3 portions/day | 26 (5.5) | 0 (0) | 6 (2.7) | 20 (17.1) |  |
|  |  | ≥4 portions/day | 16 (3.4) | 1 (0.8) | 5 (2.2) | 10 (8.5) |  |
| Vegetables ^a^ | 472 |  |  |  |  |  | <0.001 |
|  |  | “Never/seldom” | 27 (5.7) | 17 (13.4) | 7 (3,1) | 3 (2.6) |  |
|  |  | 1–3 portions/week | 98 (20.8) | 52 (40.9) | 34 (15.0) | 11 (9.4) |  |
|  |  | 4–6 portions/week | 113 (23.9) | 30 (23.6) | 71 (31.4) | 12 (10.3) |  |
|  |  | 1 portion/day | 136 (28.8) | 20 (15.7) | 75 (33.2) | 41 (35.0) |  |
|  |  | 2 portions/day | 61 (12.9) | 4 (3.1) | 31 (13.7) | 26 (22.2) |  |
|  |  | 3 portions/day | 25 (5.3) | 3 (2.4) | 4 (1.8) | 18 (15.4) |  |
|  |  | ≥4 portions/day | 12 (2.5) | 1 (0.8) | 4 (1.8) | 6 (5.1) |  |
| Physical activity ^a^ | 471 |  |  |  |  |  | <0.001 |
|  |  | ≤1/2 h/week | 12 (2.5) | 7 (5.5) | 5 (2.2) | 0 (0) |  |
|  |  | 1 h/week | 11 (2.3) | 9 (7.1) | 2 (0.9) | 0 (0) |  |
|  |  | 2 h/week | 39 (8.3) | 22 (17.3) | 16 (7.1) | 1 (0.9) |  |
|  |  | 3 h/week | 92 (19.5) | 47 (37.0) | 40 (17.7) | 5 (4.3) |  |
|  |  | ≥4 h/week | 317 (67.2) | 42 (33.1) | 163 (72.1) | 111 (94.9) |  |
| Sugary soda ^a^ | 472 |  |  |  |  |  | <0.001 |
|  |  | “Never/seldom” | 115 (24.4) | 14 (11.0) | 46 (20.4) | 55 (47.0) |  |
|  |  | 1–3 times/week | 280 (59.3) | 76 (59.8) | 147 (65.0) | 55 (47.0) |  |
|  |  | 4–6 times/week | 56 (11.9) | 26 (20.5) | 26 (11.5) | 4 (3.4) |  |
|  |  | Every day | 9 (1.9) | 5 (3.9) | 4 (1.8) | 0 (0) |  |
|  |  | 2 times/day | 4 (0.8) | 0 (0) | 3 (1.3) | 1 (0.9) |  |
|  |  | 3–4 times/day | 5 (1.0) | 3 (2.4) | 0 (0) | 2 (1.7) |  |
|  |  | >5 times/day | 3 (0.6) | 3 (2.4) | 0 (0) | 0 (0) |  |
| Sweets at school ^a^ | 472 |  |  |  |  |  | <0.001 |
|  |  | “Never/seldom” | 362 (76.7) | 78 (61.4) | 178 (78.8) | 104 (88.9) |  |
|  |  | 1–2 times/week | 98 (20.8) | 42 (33.1) | 44 (19.5) | 12 (10.3) |  |
|  |  | 3–4 times/week | 10 (2.1) | 6 (4.7) | 3 (1.3) | 1 (0.9) |  |
|  |  | “Every day” | 2 (0.4) | 1 (0.8) | 1 (0.4) | 0 (0) |  |
| Sweets at home ^a^ | 472 |  |  |  |  |  | <0.001 |
|  |  | “Never/seldom” | 32 (6.8) | 3 (2.4) | 19 (8.4) | 10 (8.5) |  |
|  |  | 1–2 times/week | 277 (58.7) | 62 (48.8) | 128 (56.6) | 85 (72.6) |  |
|  |  | 3–4 times/week | 126 (26.7) | 43 (33.9) | 62 (27.4) | 21 (17.9) |  |
|  |  | 5–6 times/week | 26 (5.5) | 13 (10.2) | 13 (5.8) | 0 (0) |  |
|  |  | “Every day” | 11 (2.3) | 6 (4.7) | 4 (1.8) | 1 (0.9) |  |
| Tobacco use | 472 |  |  |  |  |  | 0.008 |
|  |  | “Never/seldom” | 450 (95.3) | 113 (89.0) | 221 (97.8) | 114 (97.4) |  |
|  |  | 1 time/month | 8 (1.7) | 3 (2.4) | 3 (1.3) | 2 (1.7) |  |
|  |  | 2–3 times/month | 5 ( (1.1) | 3 (2.4) | 1 (0.4) | 1 (0.9) |  |
|  |  | 1–3 times/week | 2 (0.4) | 2 (1.6) | 0 (0) | 0 (0) |  |
|  |  | 4–6 times/week | 0 (0) | 0 (0) | 0 (0) | 0 (0) |  |
|  |  | “Every day” | 2 (0.4) | 1 (0.8) | 1 (0.4) | 0 (0) |  |
|  |  | “Several times/day” | 5 (1.1) | 5 (3.9) | 0 (0) | 0 (0) |  |
| Emphasis of healthy eating | 472 |  |  |  |  |  | <0.001 |
|  |  | “Very little” | 17 (3.6) | 14 (11.0) | 3 (1.3) | 0 (0) |  |
|  |  | “Little” | 51 (10.8) | 27 (21.3) | 19 (8.4) | 5 (4.3) |  |
|  |  | “Medium” | 260 (55.1) | 70 (55.1) | 138 (61.1) | 51 (43.6) |  |
|  |  | “Large” | 121 (25.6) | 11 (8.7) | 61 (27.0) | 48 (41.0) |  |
|  |  | “Very large” | 23 (4.9) | 5 (3.9) | 5 (2.2) | 13 (11.1) |  |
| Breakfast consumption | 472 |  |  |  |  |  | <0.001 |
|  |  | “Never” | 24 (5.1) | 15 (11.8) | 7 (3.1) | 2 (1.7) |  |
|  |  | 1–2 times/week | 59 (12.5) | 29 (22.8) | 24 (10.6) | 6 (5.1) |  |
|  |  | 3–4 times/week | 43 (9.1) | 19 (15.0) | 18 (8.0) | 6 (5.1) |  |
|  |  | 5–6 times/week | 51 (10.8) | 15 (11.8) | 25 (11.1) | 11 (9.4) |  |
|  |  | “Every day” | 295 (62.5) | 49 (38.6) | 152 (67.3) | 92 (78.6) |  |
| Packed lunch at school | 472 |  |  |  |  |  | <0.001 |
|  |  | “Never” | 36 (7.6) | 18 (14.2) | 14 (6.2) | 4 (3.4) |  |
|  |  | 1–2 times/week | 55 (11.7) | 27 (21.3) | 23 (10.2) | 5 (4.3) |  |
|  |  | 3–4 times/week | 106 (22.5) | 34 (26.8) | 51 (22.6) | 21 (17.9) |  |
|  |  | “Every day” | 275 (58.3) | 48 (37.8) | 138 (61.1) | 87 (74.4) |  |
| Lunch at school canteen | 472 |  |  |  |  |  | 0.004 |
|  |  | “Never” | 279 (59.1) | 64 (50.4) | 135 (59.7) | 78 (66.7) |  |
|  |  | 1–2 times/week | 145 (30.7) | 40 (31.5) | 72 (31.9) | 33 (28.2) |  |
|  |  | 3–4 times/week | 39 (8.3) | 21 (16.5) | 13 (5.8) | 5 (4.3) |  |
|  |  | “Every day” | 9 (1.9) | 2 (1.6) | 6 (2.7) | 1 (0.9) |  |
| Omega-3 supplements | 470 |  |  |  |  |  | 0.022 |
|  |  | Yes | 217 (46.1) | 45 (35.4) | 111 (49.3) | 59 (50.4) |  |
|  |  | No | 254 (53.9) | 82 (64.6) | 114 (50.7) | 58 (49.6) |  |

^a^ Indicators included in the Diet Score; ^b^ Fruit juices and smoothies not included; ^c^ Pearson’s Chi-square test (*X*^2^).

**Table S2.** Test-retest agreement of questions in the FFQ.

| **Food Groups** | ***n*** | **κ Measure of Agreement** | ***p*-Value** | **Expected Agreement in %** | **Agreement in %** |
| --- | --- | --- | --- | --- | --- |
| Fruit | 429 | 0.411 | <0.001 | 72.4 | 83.8 |
| Vegetables | 430 | 0.505 | <0.001 | 74.8 | 87.6 |
| Wholegrain content in bread | 430 | 0.509 | <0.001 | 80.2 | 90.3 |
| Seafood (dinner) | 430 | 0.478 | <0.001 | 78.6 | 88.8 |
| Seafood (spread) | 430 | 0.123 | <0.001 | 62.6 | 67.2 |
| Red meat | 430 | 0.298 | <0.001 | 80.6 | 86.6 |
| White meat | 430 | 0.383 | <0.001 | 78.9 | 87.0 |
| Dairy products | 430 | 0.469 | <0.001 | 91.1 | 84.7 |
| Sugary soda | 430 | 0.466 | <0.001 | 87.2 | 93.2 |
| Sugary sweets consumption |  |  |  |  |  |
| At school | 430 | 0.385 | <0.001 | 85.4 | 91.0 |
| At home | 430 | 0.486 | <0.001 | 80.9 | 90.2 |
| Physical activity | 430 | 0.535 | <0.001 | 80.0 | 90.7 |
| Breakfast habits | 430 | 0.696 | <0.001 | 69.0 | 90.6 |
| Packed lunch | 430 | 0.553 | <0.001 | 65.5 | 84.6 |
| Canteen lunch | 430 | 0.649 | <0.001 | 77.7 | 92.2 |
| Omega-3 supplements | 429 | 0.456 | <0.001 | 51.2 | 73.4 |
| Emphasis of healthy eating | 430 | 0.567 | <0.001 | 78.5 | 90.7 |
| Tobacco use | 430 | 0.440 | <0.001 | 95.7 | 97.6 |

FFQ: Food frequency questionnaire.
